# Learning about how public involvement strengthens HIV research as a medical student

**DOI:** 10.1186/s40900-020-00198-2

**Published:** 2020-05-07

**Authors:** Joseph Lewis

**Affiliations:** University of Exeter, Exeter, UK

**Keywords:** Public and patient involvement, Health Research methods, HIV, PROUD, Gay rights

## Abstract

**Plain English summary:**

A small medical school research project entitled ‘*PPI to strengthen clinical and population health research’* caused me to look at an entirely new field – that of Public and Patient Involvement (PPI). PPI is the development of an active partnership between patients and the public and researchers to work alongside each other not just during trial participation but, at each research step. Internet research and use of materials supplied by my tutors changed my understanding and appreciation for the field of PPI. Coming across the story surrounding gay rights campaigners and development of early PPI inspired me to investigate how it is used it modern studies. It struck me how despite its importance, PPI often went undiscussed in contemporary studies. However, one study named PROUD used and reported PPI in an exemplary fashion in order to combat issues and strengthen the trial. PROUD’s PPI improved study design, safety and advertising as well as providing reassurance in difficult times. It also improved recruitment, dissemination of information and made sure that results and future research fields were relevant. Through PROUD and also my own experiences working with a PPI group I was able to develop an understanding and appreciation for the importance PPI. I write with hope to make my learning available to others.

**Abstract:**

A research project during my third year at medical school introduced me to PPI. PPI is defined as research carried out *with* or *by* members of the public rather than *to*, *about* or *for* them. Historical gay rights activists’ involvement in research catalysed developments in PPI before it was widely recognised. A contemporary study, PROUD, used exemplary PPI contributions to tackle these issues and make the study successful. My research project was entitled *‘How did PPI contribute towards the PROUD study & what can be learnt from this?’*. This letter aims to answer this question but also include my personal reflections on my work and how I developed an understanding and appreciation for the importance PPI. PubMed and Google were examined, as well as University resources. Correspondence with PROUDs lead investigator also informed this letter. It was found that PROUD’s PPI improved study design, safety and advertising as well as providing reassurance in difficult times. It also improved recruitment, dissemination of information and made sure that results and future research fields were relevant. This allows us to understand and appreciate PPI’s role in research and the provision of healthcare. It is also important to increase discussion and learning around PPI for the future.

Dear Editors,

I write to share my experiences and reflections learning about Public and Patient Involvement (PPI) as a third-year medical student at The University of Exeter.

This academic year, as an essential part of our medical degree, every third-year student conducted a small research project from a choice of many options. One option, which intrigued me, was a project entitled ‘*PPI to strengthen clinical and population health research’*. PPI was a completely new concept to me and was something that had not been discussed at previous points during my degree. Unsure, I initially considered it to refer to the recruitment of members of the public as research participants, a view shared initially by peers also undertaking the project, but I selected the project eager to find out more. The project introduced three peers and I to PPI and our understanding was changed, now grasping and utilising INVOLVE’s definition, as research carried out *with* or *by* members of the public rather than *to*, *about* or *for* them [1]. PPI is the development of an active partnership between patients and the pubic and researchers to work alongside each other not just during trial participation but, at each research step. Through this research project I came to appreciate the value that PPI can add in producing research.

Only three other students completed this project alongside me, which raises the question whether the remaining 120 or so students in my cohort would ever be introduced to PPI during their medical school careers? In 2009 the General Medical Council (GMC) introduced guidance for medical schools on PPI in undergraduate medical education, in order to develop curriculums, admissions and assessments [2]. PPI has been going on in the background at our medical school without us knowing about it, however, the concept of PPI has not yet directly been included in compulsory teaching.

I began to research PPI using Google and resources provided by my tutors (Dr K Maguire and Dr. Cornelia Guell), I was able to develop my understanding of what PPI is and how it can be used. I found several useful articles published by the National Institute of Health Research (NIHR) (including South A [3]) and decided to investigate stigma in research further. I examined how PPI can be used to reduce stigma in mental health research, vaccination trials after the Wakefield scandal and also HIV studies. However, several papers, including that of Maguire K, et al. [4] spoke of a poignant story that struck me. During the 1980s, stigmatisation of HIV inspired gay rights activists to get involved in HIV treatment research. This catalysed significant developments in PPI before the field was widely recognised. Many people became involved in research, funding priorities shifted and even the condition’s definition was changed [4]. This historical impact of PPI inspired me to look at how contemporary HIV prevention studies use PPI to tackle similar as well as different, modern issues.

Throughout the project I conducted a small amount of my own PPI by consulting the University of Exeter’s Health and Environment Public Engagement Group (HEPE). This is a PPI group which comprises of both patients and the public from different backgrounds and experiences who live in the South West of England. I was able to present my early work at one of their quarterly meetings and they gave me valuable feedback on my ideas, as well as the relevance and interest of my work. At later stages in the project, I also received advice on abstracts and poster drafts from them through online communication. The involvement of this group in my project resulted in changes to wording and structure as well as the general messages of my work, aiming to make it more accessible to a wider audience. This further convinced me of the importance of PPI. Peers outside my research group, however, had no opportunity to consult HEPE or another PPI group when undertaking their projects. Utilising these groups could not only benefit 120 other research projects but allow students first-hand experience and a chance to learn about the concept of PPI. Hearing patient’s personal views always ignites interest amongst my peers and I, so hearing experiences of working in PPI groups and how this has affected them would be interesting. Formal teaching about how to conduct PPI in projects once qualified, for example introducing the NIHR’s Research Design Service, could help us not only be excellent doctors but provide the tools for us to be successful researchers too.

Firstly, I conducted a broad literature search on PubMed and Google including terms such as HIV and PPI. I found six HIV studies that used PPI though it was rarely reported in detail. Increasingly PPI is a funding requirement [5] however, underreporting of PPI by studies is issue that my peers and I faced when conducting our projects. It seems to be an issue that reaches far outside HIV research and makes it difficult to learn about PPI and research how it is used to improve research [6]. This problem was tackled by Staniszewska S, et al. [6] and resulted in the development on the Guidance for Reporting Improvement of Patients and the Public (GRIPP2), but more research needs to be done to see how far this has been globally adopted.

However, one study stood out not only for its use of PPI but the fact that it reported its key elements in detail. The study was called PROUD [7] and it assessed the effectiveness of the Pre-exposure prophylaxis (PrEP) drug *Truvada* for HIV prevention in men who have sex with men and trans women whilst using exemplary PPI contributions to tackle issues and make the study successful. PROUD was conducted by the Medical Research Councils Clinical Trials Unit at University College London (UCL) and was led by Professor Sheena McCormack. It was very resource rich, including a good webpage on their use of PPI and citations in several PPI systematic reviews. I used these resources and personal correspondence with Professor McCormack to complete my project entitled *‘How did PPI contribute towards the PROUD study & what can be learnt from this?’*

PROUD formed a Community Engagement Group (CEG) from the start, involving HIV charities and community groups – including the Terrance Higgins Trust [8]. During study design the CEG helped establish that, for safety, participants would have the know whether they were using PrEP or not [9], hence, they agreed to a design that randomised participants to offer of PrEP straight away or after a year of follow-up. Although recruitment was slow, the CEG assured researchers of the importance of their work [9] and released a joint statement endorsing the value of the trial, which boosted media coverage and thus recruitment [8]. Participant Involvement Meetings were also conducted [10], as vital members of the community, their options were used to improve data collection and recruitment and to inform future research priorities. Within the gay community PrEP had significant stigma due to association with sexual risk and gay media struggled to present it positively. When the results were to be released the PROUD team thought to emphasise the participants’ high HIV risk, but one meeting indicated the need for study interventions to reduce this stigma, therefore the key messages of the results were changed [9]. Meetings also identified monthly injectables as a future research priority [8]. These contributions were quantified by a recent survey of six researchers involved in PROUD, finding that they all thought the study benefitted from PPI with half of them even calling for more PPI [11]. 46 PPI participants were also surveyed and 74% thought their involvement had impacted on the study [11].

From PROUD we can learn that involving the public at the outset adds invaluable contributions to study design, safety and advertising as well as providing reassurance in difficult times. Community groups may also have greater contact with potential participants, proving useful during recruitment and dissemination of information. Using participants views can facilitate understanding of flaws in research and recruitment processes, as well as making sure the results and future research fields are relevant to the communities they affect most. We can therefore learn that, for PROUD, PPI contributions were vital to success and we should, therefore, strive to use it to produce research that is safe, well designed, feasible and relevant for the future. However, inclusion of techniques in PROUD’s method details and greater PPI reporting by studies generally using GRIPP2 (as discussed by Staniszewska S et al. [6]) would facilitate discussion and make this learning more available to others.

PPI was something that I had not experienced previously during my career but through my small research project, I gained a new understanding and appreciation of PPI’s role in research and the provision of healthcare. This understanding is something that I now wish to share with my medical school peers and your readers, as well as highlighting it as something to be more directly included in the standard undergraduate medical degree programme.

## Data Availability

Not applicable.

## Abbreviations

PPI: Public and Patient Involvement
NIHR: National Institute of Health Research
GMC: General Medical Council
HIV: Human Immunodeficiency Virus
PrEP: Pre-Exposure Prophylaxis
GRIPP2: Guidance for Reporting Improvement of Patients and the Public
UCL: University College London
HEPE: Health and Environment Public Engagement
CEG: Community Engagement Group
